# Ctr2 Links Copper Homeostasis to Polysaccharide Capsule Formation and Phagocytosis Inhibition in the Human Fungal Pathogen *Cryptococcus neoformans*

**DOI:** 10.1371/journal.pone.0012503

**Published:** 2010-09-02

**Authors:** Cheryl D. Chun, Hiten D. Madhani

**Affiliations:** Department of Biochemistry and Biophysics, University of California San Francisco, San Francisco, California, United States of America; University of Missouri-Kansas City, United States of America

## Abstract

*Cryptococcus neoformans* is a human opportunistic fungal pathogen responsible for ∼1/3 of HIV/AIDS deaths worldwide. This budding yeast expresses a polysaccharide capsule necessary for virulence. Capsule production inhibits phagocytosis by macrophages. Here we describe results that link copper homeostasis to capsule production and the inhibition of phagocytosis. Specifically, using *Agrobacterium*-mediated insertional mutagenesis, we identified an insertion in the promoter region of the putative copper transporter-encoding gene *CTR2* that results in reduced expression of *CTR2* and increased phagocytosis by murine RAW264.7 macrophages. The mutant also displayed sensitivity to copper starvation and defects in polysaccharide capsule production and melanization. These defects were all reversed by genetic correction of the promoter insertion by homologous targeting. Several melanization-defective mutants identified previously, those in the *RIM20*, *RIM101*, and *VPS25* genes, also display sensitivity to copper starvation, reduced capsule production and increased phagocytosis. Together these results indicate a previously undescribed link between copper homeostasis to polysaccharide capsule production and phagocytosis inhibition in *Cryptococcus neoformans*.

## Introduction

The fungus *Cryptococcus neoformans* is one of the leading causes of morbidity and mortality in immunocompromised patients, including organ transplant recipients on immunosuppressive therapy and AIDS patients. It is estimated that *C. neoformans* is responsible for 13–44% of the over 3 million AIDS-related deaths worldwide [1]. Although cryptococcosis is typically associated with immunodeficient individuals, a recent outbreak in the Pacific Northwest among immunocompetent individuals has stressed the importance of understanding the complex interactions of this fungal pathogen with the host immune system.

*Cryptococcus neoformans* is thought to be predominantly acquired through inhalation of spores or yeast into the lungs. Therefore, alveolar macrophages are believed to be one of the first lines of defense against cryptococcosis. Indeed, experimental evidence indicates that macrophages play an important role in host defense against cryptococcosis, especially early in infection [2], [3].

Previous studies have observed that in the absence of opsonizing agents such as complement or antibodies, *C. neoformans* is rarely taken up by macrophages, even after 24 hours of co-incubation [4], [5], [6]. This is in striking contrast to other yeast such as *Sacchromyces cerevisiae* or *Candida albicans*, or inert objects such as latex beads, all of which are taken up after less than an hour of co-incubation [7], [8], [9]. These studies suggest the possibility that *C. neoformans* may inhibit or evade unopsonized phagocytosis by macrophages.

*C. neoformans* has a number of traits known to be correlated with its virulence. These include its production of a polysaccharide capsule and its ability to synthesize the pigmented compound melanin. The production of capsule has been previously associated with inhibition of phagocytosis, although this correlation was predominantly seen in *C. neoformans* cells opsonized with serum [4], [5], [10]. Our group and others have shown a correlation between the ability of a *C. neoformans* strain to generate melanin pigment and its virulence in the host [11], [12], [13]. However, a recent screen performed by our group [6] demonstrated that while hypomelanization is correlated with defects in growth in the murine lung, a strain lacking the sole enzyme responsible for melanization, the laccase Lac1, does not display this growth defect. This suggests that melanization per se is not required for lung infectivity, but is tightly co-regulated with another trait that plays an important role in virulence. Lac1 is a diphenol oxidase that utilizes copper for its function. Thus far, *C. neoformans* has one characterized copper transporter: Ctr4. *CTR4* expression is known to be copper-regulated and is dependent on the transcription factors Cuf1 and Rim101 [14], [15].

In this study, we performed a screen using an insertional mutant library to identify genes important for inhibiting unopsonized phagocytosis. We identified a gene with homology to known copper transporters, which we have termed *CTR2*, and determined that *CTR2* falls into a class of genes that when mutated all show an increased sensitivity to copper starvation, reduced melanization and capsule formation, and increased uptake by macrophages. We hypothesize that copper uptake may play a previously uncharacterized role in capsule formation, and is important for the strong phagocytic inhibition evidenced by wild type *C. neoformans* cells.

## Results

### Screen for mutants defective in phagocytosis inhibition

We and others have previously observed that wild type *C. neoformans* cells are seldom phagocytosed by macrophages in the absence of opsonizing agents such as complement or anti-*C. neoformans* antibodies [4], [5], [6]. This is in stark contrast to the rapid uptake by macrophages of other unopsonized yeast such as *S. cerevisiae* and *Candida albicans*, suggesting that *C. neoformans* possesses mechanisms for evading phagocytic cells. Previous studies have suggested that the polysaccharide capsule surrounding the *C. neoformans* cell may contribute to immune evasion [16].

We sought to identify and characterize this putative mechanism of phagocytosis evasion by screening a mutant library generated in wild type *C. neoformans* cells for increased phagocytosis by macrophages. Through *Agrobacterium tumefaciens*-mediated insertional mutagenesis, an estimated 30,000 mutants were created and then pooled into a single library. Southern hybridization analysis indicated that the library predominantly contained strains with one site of insertion per mutant (Figure S1).

**Figure 1:**
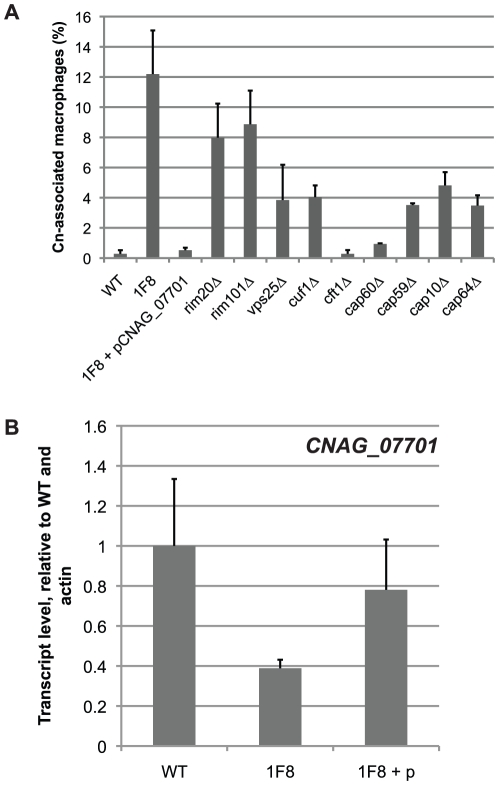
*C. neoformans mutant* strains demonstrate increased rates of unopsonized phagocytosis. (A) Mutant *C. neoformans* strains show increased rates of phagocytosis by RAW264.7 macrophages. The indicated *C. neoformans* strains were co-incubated with RAW264.7 macrophages for 24 hours. The macrophages were then washed three times with PBS to remove unphagocytosed yeast, and the percentage of macrophages with associated yeast was assayed. At least 200 macrophages were counted per strain, and each strain was performed in triplicate. Error bars denote SD. (B) RT-qPCR of *CNAG_07701* transcript in wild type, the mutant strain 1F8, and the strain 1F8 + pCNAG_07701 (1F8 + p), where the mutated promoter of *CNAG_07701* has been replaced with an intact copy. Error bars denote SD from strains grown in duplicate.

**Figure 2:**
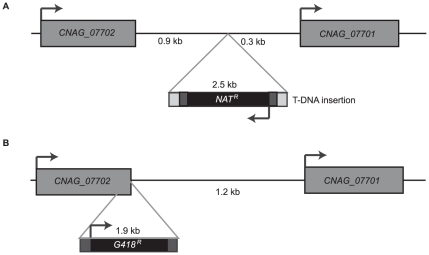
Disruption and complementation of promoter of *CNAG_07701*. (A) Schematic of *A. tumefaciens*-mediated disruption of the promoter of *CNAG_07701* in the strain 1F8. (B) Schematic of complementation of 1F8 using the full promoter of *CNAG_07701*, as in the strain 1F8 + pCNAG_07701. A construct containing the promoter of *CNAG_07701* flanked by a G418-resistance cassette was transformed into the strain 1F8, and transformants were screened for acquisition of G418-resistance and loss of nourseothricin (NAT)-resistance.

The library was screened by co-incubation in RAW264.7 macrophages for 24 hours, followed by PBS washes to remove the unphagocytosed yeast. The macrophages were then lysed to release the internalized yeast, and these yeast were cultured and plated to single colonies. Individual colonies were rescreened for increased levels of phagocytosis by RAW264.7 macrophages. This screen identified a mutant, designated strain 1F8, that leads to an increase in *C. neoformans* phagocytosis upon retesting (Figure 1A). Genomic DNA was prepared from this clone and the DNA sequence flanking the site of insertion was determined by Vectorette PCR [17]. We determined that the T-DNA insertion occurred in the promoter region of the gene *CNAG_07701*, 303 bp upstream of the start codon (Figure 2A). The disruption in pCNAG_07701 reduced *CNAG_07701* transcript levels by ∼60% (Figure 1B). When we replaced the disrupted promoter with a copy of the intact promoter region of *CNAG_07701* (Figure 2B), we observed full complementation of the phagocytosis and expression phenotypes (Figures 1A,B).

### *CNAG_07701* has homology to copper transporters

**Figure 3:**
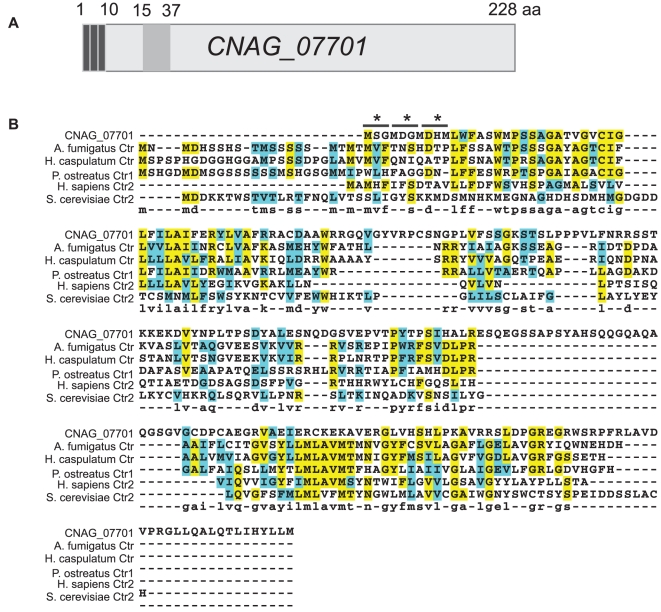
*C. neoformans* CNAG_07701 shows homology to copper transporters from other species. (A) Schematic of *CNAG_07701*. Dark grey boxes indicate presence of three MXXM motifs (residues 1–10). Light grey box indicates predicted transmembrane domain (residues 15–37). (B) ClustalW diagram comparing the sequence of *CNAG_07701* to copper transporters in *Aspergillus fumigatus, Histoplasma capsulatum, Pleurotus ostreatus, Homo sapiens*, and *Saccharomyces cerevisiae*. Asterisks (*) denote locations of MXXM motif in *CNAG_07701*.

*CNAG_07701* encodes for a protein of 228 amino acids with one predicted transmembrane domain between residues 15 and 37 (Figure 3A). It also contains three MXXM motifs at its N-terminus, where M represents a methionine and X is any amino acid. Methionine motifs such as these have been previously characterized as being important for copper uptake under copper-limiting conditions in *S. cerevisiae* [18]. A BLASTP search with the predicted protein sequence showed significant similarity between CNAG_07701 and copper transporters in *Pleurotus ostreatus* (Ctr1: 20.7% sequence identity, 25.9% sequence similarity by EMBOSS needle) [19], *Histoplasma capsulatum* (Ctr: 15.6% sequence identity, 23.3% similarity) [20], *Aspergillus fumigatus* (putative Ctr, 18.3% identity, 26.4% similarity), *Homo sapiens* (putative Ctr2: 4.7% identity and 6.5% similarity), and *S. cerevisiae* (Ctr2: 8.3% identity, 14.7% similarity) [21] (Figure 3B). Much of the similarity occurs in the first 50 residues of the N-terminus of the sequence, surrounding and including the predicted transmembrane domain, where CNAG_07701 shares 42% sequence identity with *P. ostreatus* Ctr1, 46% sequence identity with *A. fumigatus* Ctr, and 42% sequence identity with *H. capsulatum* Ctr.

**Figure 4:**
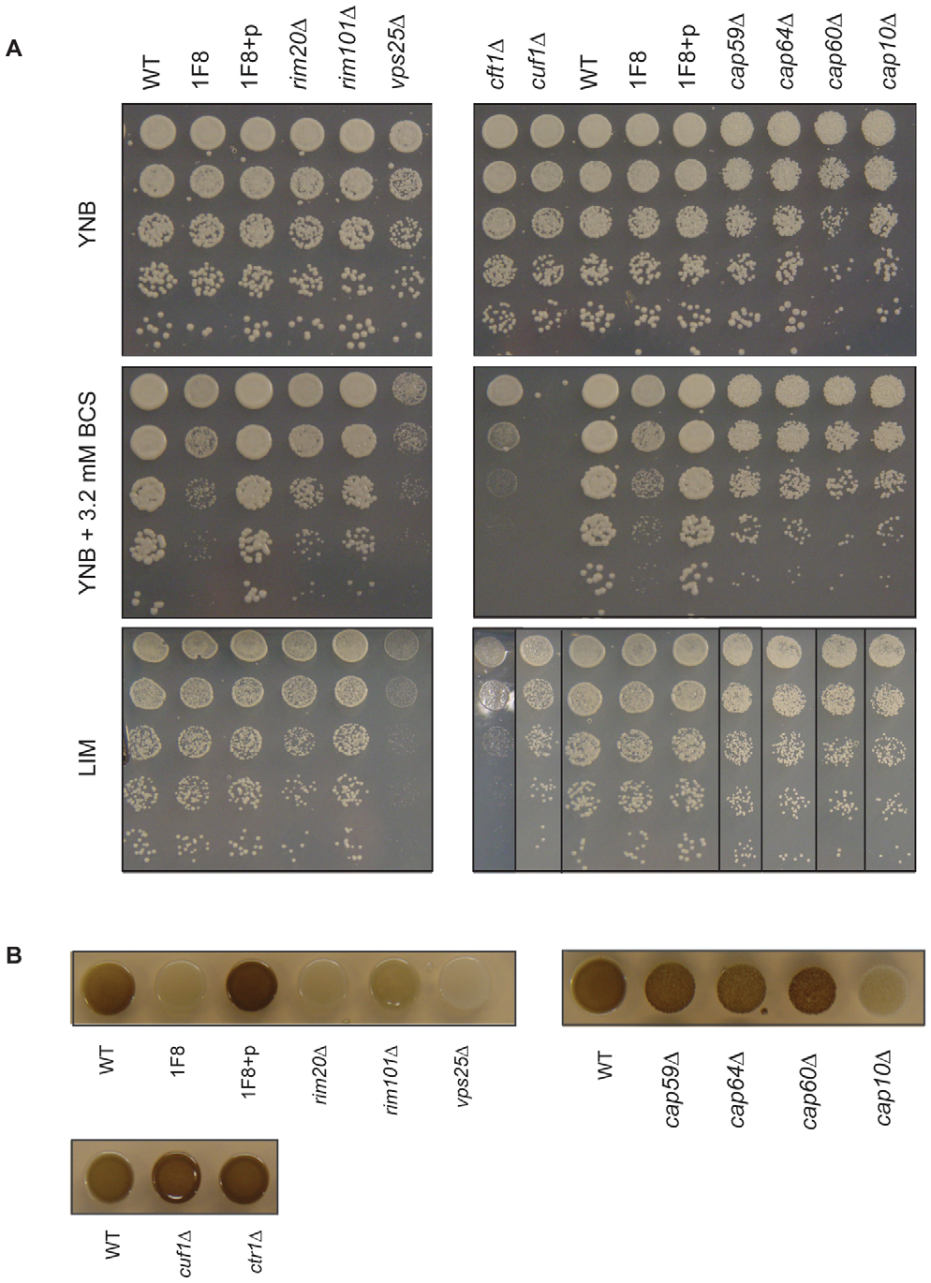
Growth and melanization defects of *C. neoformans* mutant strains. (A) Growth on YNB, YNB plates containing 3.2 mM BCS (a copper chelator, for copper-limiting conditions), and LIM (limited iron medium). The indicated strains were spotted from overnight culture in five-fold serial dilution. *LIM plate, right panel*, the images shown are all from the same plate on the same day – strains were re-arranged in image post-processing to reflect YNB and YNB + BCS plate arrangement. (B) Melanization of indicated strains. Overnight cultures were spotted on L-DOPA melanin-inducing plates.

If *CNAG_07701* encodes for a copper transporter, we hypothesized that the mutant would demonstrate a growth defect when grown in copper-limited conditions. We tested the mutant (1F8) and complemented (1F8 + pCNAG_07701) strains for sensitivity to copper starvation using the copper chelator bathocuproinedisulfonic acid (BCS). Cells were grown in YNB containing 1.6 mM BCS overnight to deplete internal copper stores, then plated in five-fold serial dilution onto YNB plates or YNB plates containing 3.2 mM BCS. Strain 1F8 showed sensitivity to growth on copper-limited medium, as hypothesized (Figure 4A). As with the phagocytosis phenotype, complementation with the intact promoter to *CNAG_07701* rescued this defect. Based on its homology to known copper transporters and the phenotypic evidence, we propose to rename *CNAG_07701* as *CTR2*.

### Ctr2 mutant is defective in capsule and melanin production

Two of the key virulence factors of *C. neoformans* are its production of the dark pigment melanin and a polysaccharide capsule. Melanin is produced by the copper-dependent enzyme laccase. We next sought to determine what role if any Ctr2 plays in production of these virulence traits. Strain 1F8 demonstrated a defect in melanization, showing much less pigment accumulation than wild type cells (Figure 4B). This is as we hypothesized, as we expected that loss of the copper transporter might have functional consequences for the copper-dependent enzyme laccase.

**Figure 5:**
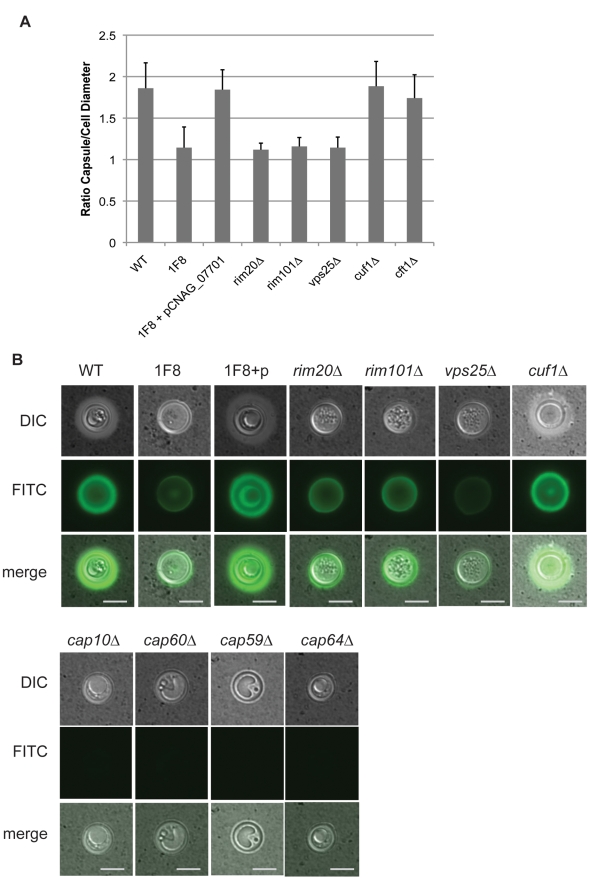
Capsule production in *C. neoformans* mutant strains. (A) Cells were grown in capsule-inducing conditions, and capsule and cell diameters were measured, and their ratio calculated, for at least thirty cells per strain. Error bars denote SD. (B) Cells from capsule-inducing conditions were stained by India ink (DIC) and anti-capsular polysaccharide antibody (FITC). Scale bar denotes 5 µm.

Strikingly, we observed a strong defect in capsule production, where strain 1F8 generates little capsule visible by India ink staining (Figure 5A). Staining with an antibody generated to one of the main components of the capsule, glucoronoxylomannan, demonstrated that the strain was able to generate capsule, but to a much lesser extent than the wild type cell (Figure 5B).

### Set of melanin mutants also show copper-starvation sensitivity

We were interested in the relationships between copper transport, capsule, melanization and phagocytosis inhibition. Our previous study had identified a number of genes that, when mutated, led to strains with lower levels of melanization [6]. We screened these 29 mutant strains to determine if their melanization defects may be coupled to defects in copper transport, capsule production and phagocytosis inhibition. From this screen, we determined that *rim20▵*, *rim101▵*, and *vps25▵* strains are sensitive to growth in copper-limited conditions (Figure 4A). Vps25 is an ESCRT-II complex subunit, responsible for trafficking vesicles to the lysosome or vacuole. Rim101 is a pH-responsive transcription factor, and Rim20 is one of its regulators [15]. Rim20 localizes to vesicles where it is thought to bring Rim101 in close proximity with an activating protease [22].

### Melanization-defective, copper starvation-sensitive mutants produce less capsule

As in the strain 1F8, capsule synthesis was reduced in *rim20▵*, *rim101▵*, and *vps25▵* strains but was still visible by immunofluorescent staining with an anti-capsule antibody (Figures 1A, 5B). This is in contrast with the acapsular strains *cap10▵*, *cap59▵*, *cap60▵*, and *cap64▵* (Figure 5B) which show no visible antibody binding. Interestingly, while the *cap▵* mutant strains displayed an overall growth defect, they did not display increased sensitivity to copper-starvation (Figure 4A). Additionally, the phagocytosis phenotypes of strains 1F8, *rim20▵* and *rim101▵* were more dramatic than the *cap▵* strains despite the different levels of capsule synthesis. These data suggest that lack of capsule does not universally confer sensitivity to copper-limitation, but that sensitivity to copper limitation is correlated with increased phagocytosis.

### *cuf1* **▵** strain phenotypes

As a control, we examined the phenotypes of a knockout of the known copper-dependent transcription factor Cuf1 [14]. As previously described, the *cuf1▵* strain demonstrated a profound growth defect in copper-limited conditions (Figure 4A), but no capsule (Figure 5A) or melanization defects (Figure 4B) [14]. As reported by Lin et al [23] and as seen in our hands, the *cuf1▵* strain does not display a melanization defect when grown on L-DOPA medium, although Lin et al demonstrated copper-dependent melanization defects on L-DOPA plates supplemented with BCS or exogenous copper. Strikingly, *cuf1▵* cells also showed increased phagocytosis by macrophages, consistent with a role in copper homeostasis in phagocytosis inhibition.

### Uncoupling of copper and iron uptake

Copper and iron are closely co-regulated in the cell, so we sought to determine if these phenotypes were attributable to defects in solely copper or in both copper and iron uptake [24], [25]. The yeast were cultured in low iron medium (LIM) for 2 days to deplete internal iron stores and then plated on LIM plates to assay for sensitivity to iron limitation (Figure 4A). As expected, a mutant in the gene for the iron permease Cft1 showed decreased growth on LIM [26], and also on copper-limited media (see Discussion). The strain *vps25▵* also showed a defect in growth in low iron, suggesting that Vps25 may play a role in both iron and copper uptake. Interestingly, strains 1F8, *rim20▵*, and *rim101▵* displayed no growth defects in LIM despite their strong phenotypes on copper-limited medium, indicating that their phenotypes are uncoupled from iron uptake (Figure 4A).

**Figure 6:**
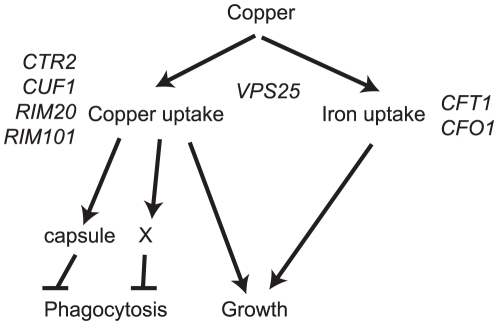
A model of the genetic basis of copper homeostasis in *C. neoformans*. Copper is required for iron uptake, through the actions of the oxidoreductase Cfo1. Copper uptake and copper-dependent growth depend on *CTR2, CUF1*, *RIM20*, *RIM101*, and *VPS25*. There is a requirement for copper for capsule biosynthesis and attachment, and for inhibition of phagocytosis, although it remains unclear if the two phenotypes are linked.

Taken together, these observations support a role for copper homeostasis in phagocytosis inhibition, and in capsule synthesis (Figure 6). It is currently unknown if the two pathways are linked.

## Discussion

We used a library of insertional mutants and a genetic screen to identify *C. neoformans* genes involved in inhibition or evasion of phagocytosis by macrophages. Through this screen, we identified the copper transporter Ctr2, which has homology to known copper transporters in other yeast. As expected, a mutant with reduced *CTR2* expression shows sensitivity to growth in copper-limited conditions. Unexpectedly, it also shows defects in capsule formation. We determined that this is not an isolated relationship – strains with knockouts in *RIM20*, *RIM101* and *VPS25* are also sensitive to growth in copper-limited conditions and show decreased capsule production. We believe this points to a new, previously uncharacterized links between copper uptake and capsule synthesis, and between copper uptake and anti-phagocytic behavior (Figure 6).

It remains uncertain if the reduced capsule production is directly responsible for the reduction in anti-phagocytic behavior. It is striking that the strains 1F8, *rim20▵*, *rim101▵* and *cuf1▵* display more phagocytosis than *cap10▵*, *cap59▵*, *cap60▵* and *cap64▵* strains, despite generating more capsule, suggesting that the relationship between capsule and phagocytosis inhibition is not a linear one. This may suggest copper-dependent synthesis of an inhibitory factor, or a copper-dependent modification of the capsule.

This study is the first to link sensitivity to growth on copper-limited conditions with capsule production in *C. neoformans*. It is interesting that the ESCRT-II component Vps25 emerged as important for capsule formation and copper uptake, as it is important for vesicular trafficking to the vacuole. Previous studies have shown that the copper transporters Ctr2 (*S. cerevisiae*) and Ctr6 (*S. pombe*) are localized to the vacuole, which may serve as a site for copper storage and mobilization [27], [28]. In *S. cerevisiae*, a recent screen identified the strain *vps25▵* as well as strains mutated in other members of the ESCRT-II and ESCRT-III complexes as sensitive to copper overload, confirming a role for Vps25 and the ESCRT machinery in copper homeostasis [29]. Additionally, studies have suggested that capsule formation occurs within the cell and is transported by vesicles, as evidenced by anti-GXM antibody staining of vesicles in a mutant defective for secretion [30]. It is therefore possible that the defects of the *vps25▵* strain in both capsule synthesis and copper homeostasis may be attributable to a requirement in both for proper vesicle formation.

Cft1 is a homolog of the *S. cerevisiae* high affinity iron transporter Ftr1. Ftr1 complexes with the multicopper ferroxidase Fet3, which utilizes copper to oxidize Fe^2+^ to Fe^3+^ for transport by Ftr1 into the cell. Therefore, depletion of copper is known to render cells defective in iron uptake, although there is no known reciprocal requirement for iron in copper uptake in yeast [31]. As copper uptake is presumed to be normal in *C. neoformans cft1▵* cells, it is therefore expected that the *cft1▵* strain would not display a phagocytosis phenotype.

It is currently unknown what factors regulate *CTR2* transcriptional expression. Previous studies have shown that *CTR4* is regulated by Cuf1 and Rim101 [14], [15]; it remains to be seen if *CTR2* is similarly regulated. However, O'Meara et al [15] performed microarrays comparing gene expression in a *rim101▵* strain to wild type and did not identify *CTR2* in their analysis, suggesting that its expression is Rim101-independent. In our hands, the strain *rim101Δ* displayed some surprising phenotypes that were unexpected based on the studies performed by O'Meara et al. Specifically, we observed no growth defect in LIM and increased levels of phagocytosis. O'Meara et al observed a mild growth defect for strain *rim101Δ* when grown in liquid LIM culture, but noted that the mutant did eventually reach saturation phase. Our LIM assay utilized cultures at saturation in LIM prior to spotting onto LIM plates, and it is possible that in those conditions there are Rim101-independent adaptations to growth in limited iron that allow for robust growth on LIM plates. In addition, while we saw increased levels of phagocytic uptake of *rim20Δ* and *rim101Δ* strains, O'Meara et al reported no change in the phagocytic index of the *rim101Δ* strain when compared to wild type. This is likely due to their use of opsonization (personal communication), as we have observed robust uptake of wild type and mutant strains when opsonized with anti-capsular antibodies that masks subtler phagocytosis phenotypes (data not shown). We were able to confirm their observation that *rim20Δ* and *rim101Δ* cells do not display capsule by India ink staining when cultured in DMEM, at 37°C with 5% CO_2_. This is in contrast with our earlier findings [6], where we reported no capsule phenotypes for these strains or the strain *vps25Δ*. This is due to a difference in the conditions used to induce capsule: in our previous studies, capsule-induction was performed in 10% Saboraud dextrose medium, and in these conditions, *rim20Δ* and *rim101Δ* strains generate capsule on levels comparable to wild type (Figure S2). This suggests that there are different signaling pathways that feed into capsule production, and that Rim101 is required for capsule attachment in one condition but not the other. This is to our knowledge the first evidence for transcriptional regulation of different pathways for capsule synthesis in response to different stimuli.

This study links for the first time capsule biogenesis and phagocytosis inhibition to copper homeostasis in *C. neoformans*. Future studies are required to determine the exact mechanisms through which copper affects these two processes.

## Materials and Methods

### Gene nomenclature

Genes were identified using annotation from the H99 sequence from the Broad Institute (http://www.broadinstitute.org/annotation/genome/cryptococcus_neoformans/MultiHome.html) and from our own annotation of the H99 sequence (http://cryptogenome.ucsf.edu). Gene annotations from the Broad are designated by their nomenclature “CNAG_####”, while our own annotations are designated “CDS_####”.

### Strains and media

*Agrobacterium tumefaciens* strain C601 containing the plasmid pYCC710 (which has the nourseothricin resistance gene [NAT]) was a gift from June Kwon-Chung [32], and was maintained on LB agar plus 100 µg/ml kanamycin. For cryptococcal transformations, C601 was grown in agrobacterium minimal medium [33] with 100 µg/ml kanamycin or in induction medium [34] with 100 µg/ml kanamycin and 200 µM acetosyringone. *C. neoformans* strains relevant to this study are listed in Table 1. All strains were constructed in the H99 strain background. *C. neoformans* was routinely grown on YPAD medium (1% yeast extract, 2% Bacto-peptone, 2% glucose, 0.015% L-tryptophan, 0.004% adenine). Selective medium contained nourseothricin (Werner BioAgents, Jena-Cospeda, Germany, 0.1 mg/ml) or G418 (VWR, 0.2 mg/ml). The strain *cuf1Δ* was generated by knocking out the gene CDS_2478 using biolistic transformation as previously described [6]. RAW264.7 macrophages were maintained in RAW medium (Dulbelco's Modified Eagle Medium [DMEM] with 4.5 g/L glucose, 20 mM HEPES/NaOH buffer [pH 7.4], 20 mM glutamine, 10% heat-inactivated fetal bovine serum [FBS] at 37°C with 5% CO_2_, and were used between passages 3–15. For phagocytosis assays, macrophages were plated in DMEM with 4.5 g/L glucose.

**Table 1:** Strains used in this study.

Genotype	Strain number	reference
WT (H99)	CM18	gift from J.K. Lodge
*ctr2*	CM199 (1F8)	this study
*ctr2::CTR2*	CM200 (1F8 + p)	this study
*rim20Δ*	D825	Liu et al (2008)
*vps25Δ*	D1222	Liu et al (2008)
*rim101Δ*	D1258	Liu et al (2008)
*cft1Δ*	D1785	Liu et al (2008)
*cap10Δ*	D629	Liu et al (2008)
*cap59Δ*	D45	Liu et al (2008)
*cap60Δ*	D42	Liu et al (2008)
*cap64Δ*	D679	Liu et al (2008)
*cuf1Δ*	D1882	this study

### *A. tumefaciens*-mediated transformation of *C. neoformans*

Transformation was carried out as in McClelland et al (2005) with minor changes: prior to mixing strain C601 with H99, the OD_600_ of C601 was adjusted to 0.5 and H99 was adjusted to 5.85. Equal aliquots of C601 and H99 were mixed together. 400 µl of C601+H99 were plated onto 0.45 µm Biodyne® A membranes (PALL Life Sciences, Cat. no. 601012) placed on induction medium agar plates containing 200 µM acetosyringone. The plates were incubated at 25°C for 3 days. The membranes were then transferred to YPAD plates containing 0.1 mg/ml nourseothricin and 200 µM cefotaxime. These plates were then incubated at 30°C for two days until *C. neoformans* growth was seen. The membranes were then washed with PBS to remove the *C. neoformans* cells and the cells from all membranes were pooled together. The resulting library was washed three times with PBS, before being frozen down in 15% glycerol. We estimated ∼30,000 transformants were generated in this library.

### Complementation of 1F8

Fusion PCR as described elsewhere [6] was used to generate a construct containing the G418 resistance marker flanked by 1 kb of the 3′ end of the gene CNAG_07702 upstream of *CNAG_07701* and flanked by the promoter of *CTR2* (*CNAG_07701*), spanning the region from the start codon of *CNAG_07701* to the stop codon of the upstream gene CNAG_07702. Through biolistic transformation, this construct was introduced into the strain 1F8. Transformants were screened on YPAD plates containing G418, then replica-plated onto YPAD plates containing nourseothricin to assay for sensitivity to nourseothricin and hence loss of the T-DNA insertion.

### Phagocytosis Screen with Insertional Mutant Library

2×10^7^ RAW264.7 macrophages were seeded into 15 cm tissue culture dishes (Corning) in 20 ml DMEM medium and allowed to adhere overnight. *C. neoformans* cells from overnight cultures grown in YPAD medium with 200 µm cefotaxime were washed three times with PBS then added to the RAW264.7 macrophages in 20 ml fresh DMEM at an MOI of 10∶1. Following 24 hours co-incubation, the macrophages were washed three times with PBS to remove unphagocytosed yeast, then lysed with 0.01% SDS. Cell lysis was confirmed visually on a light microscope. The lysed cells were collected and washed three times in PBS prior to resuspension in YPAD medium with 200 µM cefotaxime. The harvested yeast were then plated on YPAD agar plates containing nourseothricin and 200 µM cefotaxime. Colonies that grew up were picked and individually assayed for rates of phagocytosis.

### Phagocytosis assay

RAW264.7 macrophages (2×10^4^/well) were seeded into 96-well tissue-culture treated plates (Corning) in DMEM medium and allowed to adhere overnight. *C. neoformans* cells grown in YPAD medium were washed three times with PBS then resuspended to a density of 5×10^6^ cells/ml in PBS, and 10 µl (5×10^4^ cells) were co-incubated with the RAW264.7 macrophages in 200 µl fresh DMEM. Following 24 hours co-incubation, the macrophages were washed three times with PBS to remove unphagocytosed yeast, then fixed with 1% formaldehyde/PBS. Percentage of cell-associated macrophages was determined by counting the number of macrophages with yeast internalized or associated with their cell surface, divided by the number of macrophages counted. At least 200 macrophages were assayed per well, and each strain was tested in triplicate.

### Nucleic acid protocols

For the Southern Blot of the insertional mutant library, genomic DNA was extracted using CTAB phenol-chloroform extraction and digested with BamHI and XhoI restriction enzymes, separated on a 0.8% gel, and blotted onto Hybond N+ membrane (Amersham Biosciences). Radiolabeled probes were generated by amplifying the nourseothricin-resistance cassette, including the ACT1 promoter and TRP terminator. To identify the site of mutagenesis in the strain 1F8, genomic regions flanking the T-DNA insertion were amplified using Vectorette PCR [17]. Briefly, genomic DNA was isolated and digested with RsaI. A bubble anchor primer was annealed with T4 ligase. The ligated fragments were amplified by PCR using a primer specific to the T-DNA insertion (NAT-BUB-3′) and a primer specific to the bubble anchor (P224-3). The reaction was resolved on a 2% agarose gel, the amplified bands were excised, purified, and cloned into a TOPO vector prior to transformation into TOP10 *E. coli* cells (Invitrogen). Plasmid DNA from positive transformants was isolated and sequenced. For RT-qPCR, RNA was extracted from OD_600_ = 50 cells grown in 20 ml DMEM for 24 hours at 37°C with 5% CO_2_. The RNA was DNaseI-treated (Roche) and reverse transcribed with random nonamers and oligo(dT) primers to prime. The RNA was then digested from the cDNA with RNaseH prior to qPCR using primers against the *CNAG_07701* transcript (primers C2629/C2360) and against *ACT1* (C1208/C1209). For each primer set, standard curves were generated using five-fold sequential dilutions of cDNA to account for differences in priming efficiencies. For each sample, values obtained were normalized to the levels of actin (*ACT1*).

### Sequence Analysis

The predicted protein sequence of *CNAG_07701* was obtained from the Broad Institute H99 annotation. SMART sequence analysis (http://smart.embl-heidelberg.de) predicted a transmembrane domain in residues 15–37. ClustalW analysis was performed with the sequences of *C. neoformans CNAG_07701*, *H. capsulatum* Ctr (ABF22675.1), *Pleurotus ostreatus* Ctr1 (CAG29170.1), *A. fumigatus* hypothetical copper transporter Ctr (XP_747796.1), *S. cerevisiae* Ctr2, and *H. sapiens* Ctr2.

### Sensitivity to Copper Starvation

*C. neoformans* strains were grown in YNB medium (0.15% yeast nitrogen base w/o amino acids, w/o dextrose, w/o ammonium sulfate [Bio101], 75 mM ammonium sulfate, 2% glucose) containing 1.6 mM bathrocuproine disulfonic acid (BCS, Sigma) overnight. The cultures were diluted in water to an OD_600_ = 0.6, then five-fold serially diluted in water prior to spotting on YNB plates or YNB plates containing 3.2 mM BCS. Plates were incubated at 37°C for three days.

### Sensitivity to Iron Starvation

*C. neoformans* strains were grown in 96-well deep-pocket plates without shaking in 0.6 ml LIM medium [35] for two days to deplete intracellular iron stores. The cultures were then diluted in water to an OD_600_ = 0.6, then five-fold serially diluted in water prior to spotting on 0.5X LIM plates. Plates were incubated at 37°C for three days.

### Melanin assay

From overnight culture, *C. neoformans* cells were diluted to OD_600_ = 0.6 in water then spotted onto melanin-inducing plates containing L-DOPA (L-dihydroxyphenylalanine, Sigma, 100 mg l^−1^) and grown for three days at 37°C [36].

### Capsule assays

From overnight YPAD culture, 5×10^6^ *C. neoformans* cells were washed with PBS prior to incubation in 0.5 ml DMEM in 24-well tissue culture-treated dishes (BD Biosciences) for 2 days, with 5% CO_2_ at 37°C to induce capsule formation. The cells were harvested, fixed with 1% formaldehyde/PBS, then washed three times with PBS. For immunofluorescent imaging of capsule, the cells were then incubated with a previously described monoclonal antibody against the main capsular polysaccharide glucoronoxylomannan (mAb 339), for one hour at 37°C [37]. The cells were then washed three times with PBS and incubated with FITC-conjugated donkey anti-mouse antibody for one hour at room temperature in the dark. The cells were washed with PBS prior to resuspension with India ink for imaging. Images were taken using an Axiovert 200 M (Zeiss) microscope running Axiovision software. Exposure times for the FITC channel were kept constant at 500 ms for all strains tested. Capsule measurements were performed on at least thirty cells of each strain.

## Supporting Information

Figure S1 Genomic DNA from wild type (WT) and eight clones (1–8) from the mutant library generated by *A. tumefaciens*-mediated transformation were probed with radiolabeled probe to nourseothricin resistance cassette, which consists of the NAT gene flanked by the ACT1 promoter and TRP terminator.
(0.77 MB EPS)

Figure S2 Capsule production by rim20 and rim101strains grown in 10% Saboraud dextrose medium. *C. neoformans* strains were grown overnight in Saboraud dextrose medium and then diluted in 10% Saboraud dextrose for capsule induction. The cultures were grown for two days at 30°C prior to India ink staining.
(0.76 MB EPS)

## References

[1] Park BJ, Wannemuehler KA, Marston BJ, Govender N, Pappas PG (2009). Estimation of the current global burden of cryptococcal meningitis among persons living with HIV/AIDS.. AIDS.

[2] Monga DP (1981). Role of macrophages in resistance of mice to experimental cryptococcosis.. Infect Immun.

[3] Osterholzer JJ, Milam JE, Chen GH, Toews GB, Huffnagle GB (2009). Role of dendritic cells and alveolar macrophages in regulating early host defense against pulmonary infection with Cryptococcus neoformans.. Infect Immun.

[4] Zaragoza O, Taborda CP, Casadevall A (2003). The efficacy of complement-mediated phagocytosis of *Cryptococcus neoformans* is dependent on the location of C3 in the polysaccharide capsule and involves both direct and indirect C3-mediated interactions.. Eur J Immunol.

[5] Levitz SM, DiBenedetto DJ (1989). Paradoxical role of capsule in murine bronchoalveolar macrophage-mediated killing of *Cryptococcus neoformans*.. J Immunol.

[6] Liu OW, Chun CD, Chow ED, Chen C, Madhani HD (2008). Systematic genetic analysis of virulence in the human fungal pathogen *Cryptococcus neoformans*.. Cell.

[7] Lohse MB, Johnson AD (2008). Differential phagocytosis of white versus opaque *Candida albicans* by *Drosophila* and mouse phagocytes.. PLoS One.

[8] Tejle K, Magnusson KE, Rasmusson B (2002). Phagocytosis and phagosome maturation are regulated by calcium in J774 macrophages interacting with unopsonized prey.. Biosci Rep.

[9] Parod RJ, Brain JD (1983). Uptake of latex particles by macrophages: characterization using flow cytometry.. Am J Physiol.

[10] Bulmer GS, Sans MD (1968). *Cryptococcus neoformans*. 3. Inhibition of phagocytosis.. J Bacteriol.

[11] Noverr MC, Williamson PR, Fajardo RS, Huffnagle GB (2004). CNLAC1 is required for extrapulmonary dissemination of *Cryptococcus neoformans* but not pulmonary persistence.. Infect Immun.

[12] Kwon-Chung KJ, Polacheck I, Popkin TJ (1982). Melanin-lacking mutants of *Cryptococcus neoformans* and their virulence for mice.. J Bacteriol.

[13] Kwon-Chung KJ, Rhodes JC (1986). Encapsulation and melanin formation as indicators of virulence in *Cryptococcus neoformans*.. Infect Immun.

[14] Waterman SR, Hacham M, Hu G, Zhu X, Park YD (2007). Role of a CUF1/CTR4 copper regulatory axis in the virulence of *Cryptococcus neoformans*.. J Clin Invest.

[15] O'Meara TR, Norton D, Price MS, Hay C, Clements MF (2010). Interaction of *Cryptococcus neoformans* Rim101 and protein kinase A regulates capsule.. PLoS Pathog.

[16] Doering TL (2009). How sweet it is! Cell wall biogenesis and polysaccharide capsule formation in *Cryptococcus neoformans*.. Annu Rev Microbiol.

[17] Arnold C, Hodgson IJ (1991). Vectorette PCR: a novel approach to genomic walking.. PCR Methods Appl.

[18] Puig S, Lee J, Lau M, Thiele DJ (2002). Biochemical and genetic analyses of yeast and human high affinity copper transporters suggest a conserved mechanism for copper uptake.. J Biol Chem.

[19] Penas MM, Azparren G, Dominguez A, Sommer H, Ramirez L (2005). Identification and functional characterisation of *ctr1*, a *Pleurotus ostreatus* gene coding for a copper transporter.. Mol Genet Genomics.

[20] Gebhart D, Bahrami AK, Sil A (2006). Identification of a copper-inducible promoter for use in ectopic expression in the fungal pathogen *Histoplasma capsulatum.*. Eukaryot Cell.

[21] Kampfenkel K, Kushnir S, Babiychuk E, Inze D, Van Montagu M (1995). Molecular characterization of a putative *Arabidopsis thaliana* copper transporter and its yeast homologue.. J Biol Chem.

[22] Boysen JH, Mitchell AP (2006). Control of Bro1-domain protein Rim20 localization by external pH, ESCRT machinery, and the *Saccharomyces cerevisiae* Rim101 pathway.. Mol Biol Cell.

[23] Lin X, Huang JC, Mitchell TG, Heitman J (2006). Virulence attributes and hyphal growth of *C. neoformans* are quantitative traits and the MATalpha allele enhances filamentation.. PLoS Genet.

[24] Nyhus KJ, Jacobson ES (1999). Genetic and physiologic characterization of ferric/cupric reductase constitutive mutants of *Cryptococcus neoformans*.. Infect Immun.

[25] Jung WH, Kronstad JW (2008). Iron and fungal pathogenesis: a case study with *Cryptococcus neoformans*.. Cell Microbiol.

[26] Jung WH, Sham A, Lian T, Singh A, Kosman DJ (2008). Iron source preference and regulation of iron uptake in *Cryptococcus neoformans*.. PLoS Pathog.

[27] Portnoy ME, Schmidt PJ, Rogers RS, Culotta VC (2001). Metal transporters that contribute copper to metallochaperones in *Saccharomyces cerevisiae*.. Mol Genet Genomics.

[28] Bellemare DR, Shaner L, Morano KA, Beaudoin J, Langlois R (2002). Ctr6, a vacuolar membrane copper transporter in *Schizosaccharomyces pombe.*. J Biol Chem.

[29] Jo WJ, Loguinov A, Chang M, Wintz H, Nislow C (2008). Identification of genes involved in the toxic response of *Saccharomyces cerevisiae* against iron and copper overload by parallel analysis of deletion mutants.. Toxicol Sci.

[30] Yoneda A, Doering TL (2006). A eukaryotic capsular polysaccharide is synthesized intracellularly and secreted via exocytosis.. Mol Biol Cell.

[31] Szczypka MS, Zhu Z, Silar P, Thiele DJ (1997). *Saccharomyces cerevisiae* mutants altered in vacuole function are defective in copper detoxification and iron-responsive gene transcription.. Yeast.

[32] McClelland CM, Chang YC, Kwon-Chung KJ (2005). High frequency transformation of *Cryptococcus neoformans* and *Cryptococcus gattii* by *Agrobacterium tumefaciens*.. Fungal Genetics and Biology.

[33] Hooykaas PJJ, Roobol C, Schilperoort RA (1979). Regulation of the transfer of Ti plasmids of *Agrobacterium tumefaciens.*. J Gen Microbiol.

[34] Melchers LS, Regensburg-Tuink AJ, Schilperoort RA, Hooykaas PJ (1989). Specificity of signal molecules in the activation of *Agrobacterium* virulence gene expression.. Mol Microbiol.

[35] Zaragoza O, Casadevall A (2004). Experimental modulation of capsule size in *Cryptococcus neoformans*.. Biol Proced Online.

[36] Chun CD, Madhani HD, Abelson J, Simon M (2010). Applying Genetics and Molecular Biology to the Study of the Human Pathogen *Cryptococcus neoformans*.. Methods in Enzymology.

[37] Belay T, Cherniak R, Kozel TR, Casadevall A (1997). Reactivity patterns and epitope specificities of anti-*Cryptococcus neoformans* monoclonal antibodies by enzyme-linked immunosorbent assay and dot enzyme assay.. Infect Immun.

